# Central sensitization inventory scores correlate with pain at rest in patients with hip osteoarthritis: a retrospective study

**DOI:** 10.1186/s12891-020-03630-6

**Published:** 2020-09-05

**Authors:** Yoshihisa Ohashi, Kensuke Fukushima, Gen Inoue, Kentaro Uchida, Tomohisa Koyama, Maho Tsuchiya, Katsufumi Uchiyama, Naonobu Takahira, Masashi Takaso

**Affiliations:** 1 Department of Orthopedic Surgery, Kitasato University School of Medicine, 1-15-1 Minami-ku, Kitasato, Sagamihara City, Kanagawa 252-0374 Japan; 2 Department of Rehabilitation, Kitasato University School of Allied Health Sciences, 1-15-1 Minami-ku, Kitasato, Sagamihara City, Kanagawa 252-0374 Japan

**Keywords:** Hip osteoarthritis, Central sensitization, Central sensitization inventory, Pain at rest, Hip pain, Total hip Arthroplasty

## Abstract

**Background:**

Patients with persistent pain due to osteoarthritis (OA) complain of multiple symptoms that cannot be explained solely by structural changes. A poor correlation exists between structural and inflammatory changes in OA and pain levels. Central sensitization (CS) has been identified as a factor that induces chronic pain in patients with OA. Although it is important to identify osteoarthritis patients with CS components, the prevalence and characteristics of CS, especially those in patients with hip OA, are not well understood. Thus, we aimed to determine the prevalence and characteristics of CS in patients with hip OA, in this study.

**Methods:**

The CS Inventory (CSI), used as a non-invasive routine clinical tool to evaluate the presence of CS 1 month before surgery in 100 patients with hip OA, was measured at our outpatient clinic, and the data were retrospectively reviewed. We determined the number of patients with a CSI score of 40 points or higher and assessed the relationships between the CSI score and clinical factors (including age, duration of hip pain, degree pain at rest and on activity, by using the visual analogue scale [VAS] and the Harris Hip Score) using the Spearman’s correlation coefficient.

**Results:**

The mean age of participants was 63.9 ± 11.6 years, and there were 15 men and 85 women. All patients had hip OA, categorised as advanced and terminal stage (Tönnis grade 2–3) on preoperative plain radiography. The mean duration of hip pain was 4.2 ± 4.4 years. The mean CSI score was 19.5 ± 11.3 and 5 (5.0%) of the patients had a score of 40 or more points. CSI scores correlated significantly only with VAS pain at rest (*r* = 0.348, *P* < 0.001).

**Conclusion:**

In this study, 1 out of every 20 hip OA patients had CS components. CSI scores were significantly correlated with pain at rest in hip OApatients. CS approaches to hip OA may be one of the treatment options for pain at rest.

## Background

Osteoarthritis (OA) is a heterogeneous disease, characterised by progressive cartilage loss, subchondral bone remodelling, osteophyte formation, and synovial inflammation, with resultant joint pain and increasing functional disability. Patients with persistent pain due to OA complain of multiple symptoms that cannot be explained solely by structural changes. It has been reported that there is a poor correlation between structural and inflammatory changes in OA and pain levels [1]. Although the determinants of pain in OA are poorly understood, they are believed to involve multiple interactive pathways that are best explained by a biopsychosocial framework, which includes biological, psychological, and social factors [2, 3].

In 2011, the International Association for the Study of Pain defined central sensitization (CS) as increased responsiveness of nociceptive neurons in the central nervous system to normal or subthreshold afferent input. CS results from persistent, intense nociceptor stimulation that triggers changes in the central pain transmitting neurons, leading to alterations in pain presentation and perception, and centrally mediated symptoms, such as fatigue and mood disorders [4]. Over the last several decades, many authors have reported CS as one of the mechanisms underlying various chronic pain disorders, including headache, whiplash pain, musculoskeletal pain, low back pain, visceral pain, vulvodynia, prostatitis, etc [5]. CS has also been reported as a chronic pain factor in patients with OA [6, 7]. Patients with chronic pain with CS components have been reported to be resistant to conservative treatment, including traditional physiotherapy and pain medication [8]. In recent years, some treatments have been reported for OA patients with CS components [8–11]. While it is important to identify OA patients with CS components, the prevalence and characteristics of CS, especially in patients with hip OA, are not well understood.

The CS Inventory (CSI) was developed as a comprehensive screening tool with high reliability and reproducibility to identify the existence of a CS component [12, 13]. The CSI comprises of two parts: part A, designed to evaluate symptoms associated with CS, comprises 25 self-reported items on somatic and emotional symptoms, scored from 0 to 100 points, with 0 and 100 being the best and worst scores, respectively. Each item was graded on a 5-point Likert scale (0: never, 1: rarely, 2: sometimes, 3: often, 4: always). Part B screens for previous diagnoses of one or more specific disorders, including seven separate CS syndromes (CSSs) (e.g.*,* fibromyalgia, chronic fatigue syndrome, temporomandibular joint disorder, irritable bowel syndrome, migraine or tension headaches, multiple chemical sensitivities, and restless leg syndrome). CS involvement is strongly suggested in these seven disorders, and CSS was proposed as a comprehensive disease concept [14, 15]. Although some studies have reported that the CSI revealed the presence of CS components in knee OA [11, 16], such investigations in hip OA were not determined.

Here, we determined the prevalence and characteristics of CS in patients with hip OA using CSI in this study.

## Methods

### Ethics

Ethical approval was obtained from our Institutional Review Board (approval number: B20–096), and the study was performed in accordance with the ethical standards laid down in the 1964 Declaration of Helsinki and its later amendments. The requirement for informed consent was waived because of the retrospective study design.

### Participants

As accumulating evidence indicates the importance of evaluating the CS components in OA pain, CSI was used as a non-invasive routine clinical tool to evaluate the presence of CS in patients with hip OA, 1 month before surgery, at our outpatient. The data of a total of 100 consecutive patients diagnosed with hip OA who were scheduled to undergo total hip arthroplasty between November 2018 and September 2019 were retrospectively included in this study. Patients who were not diagnosed with hip OA and/or previously underwent a hip surgery on the same laterality were excluded.

### Assessment of CS involvement

**Fig. 1:**
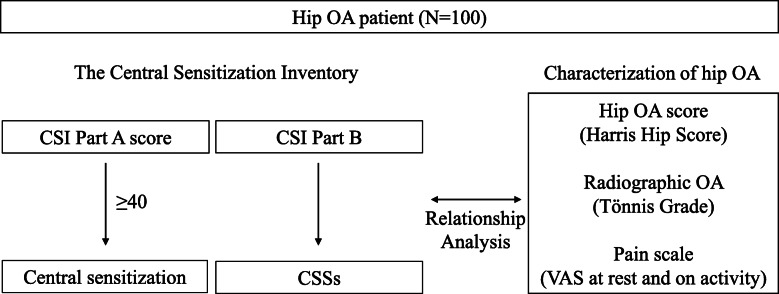
Scheme of study protocol. One hundred hip osteoarthritis (OA) patients were assessed using the Central Sensitization Inventory. The relationship between CSI Parts A and B and clinical characteristics including hip OA score (Harris Hip Score), radiographic OA grade (Tönnis grade), and pain score (visual analogue scale at rest and on activity) were also evaluated. Abbreviations: OA: Osteoarthritis; CSI: Central Sensitization Inventory; CSSs: Central Sensitization Syndromes; HHS: Harris Hip Score; VAS: visual analogue scale

To determine the existence of CS components, patients were assessed using the CSI. An explanation of the Japanese version of the CSI (both parts A and B) were provided to the patients, and the patients completed the CSI by themselves. Tanaka et al. reported that the Japanese version of the CSI is a useful and psychometrically sound tool, comparable to the English version, for assessing CSSs in Japanese patients with musculoskeletal disorders [17]. The CSI Part A score was divided into five categories with increasing severity: subclinical (0–29), mild (30–39), moderate (40–49), severe (50–59), and extreme (60–100) [18]. We determined the number of patients with a score of 40 points or higher based on previous studies as shown in Fig. 1 [13, 16, 19]. The CSI Part B was also completed to evaluate the prevalence of CSSs in the patients. The prevalence of hip OA patients with one or more CSSs was investigated.

### Relationship analysis between CSI score and clinical characteristics

To analyse the relationship between clinical scores (CSI score and the Harris Hip Score [HHS]) and clinical characteristics (age, sex, and clinical assessments [the duration of hip pain in years, severity of hip pain at rest and on activity), data were collected preoperatively at an outpatient clinic 1 month before surgery.

Radiographic OA was graded as 0 (no changes); 1 (slight narrowing of the joint space, slight lipping at the joint margin, and slight sclerosis of the femoral head or acetabulum); 2 (the presence of small bony cysts, further narrowing of the joint space, and moderate loss of femoral head sphericity); and 3 (the most severe, and indicates large cysts, severe narrowing of the joint space, severe femoral head deformity, and avascular necrosis) using the Tönnis classification. The severity of OA-related hip pain at rest and on activity was assessed using an 11-point visual analogue scale (VAS: 0 = no pain, 10 = worst possible pain). HHS, with a maximum of 100 points, is used to assesses the hip function [scores range from 0 (worse disability) to 100 (less disability)] and includes the following domains: pain, function, deformity, and motion.

### Statistical analysis

Results are expressed as the mean and the standard deviation of the mean unless otherwise indicated. A sample size calculation was performed based on the study’s main objective, which was to evaluate the correlation between CSI scores and significant variables in all patients. The minimum power level was set at 0.80. It was determined that we needed to include 97 patients to satisfy the expected effect size. Therefore, our sample size of 100 patients was deemed adequate. Continuous variables were calculated using the Mann-Whitney U test and categorical variables were calculated using the chi-square test. Correlations between the CSI score and age, duration of hip pain, VAS pain at rest, VAS pain on activity, and HHS in all patients were evaluated using the Spearman’s correlation coefficient. Correlation coefficient is represented by r. All statistical analyses were performed using Statistical Package for the Social Sciences software (version 25.0, IBM, NY, USA). *P* values < 0.05 were considered to indicate statistical significance.

## Results

### Patient demographic information

Patient demographics and clinical assessments are summarised in Table 1. The mean age was 63.9 ± 11.6 years, and there were 15 men and 85 women. All patients had hip OA, categorised as advanced and terminal stage (Tönnis grade 2–3) on preoperative plain radiography. The mean duration of hip pain was 4.2 ± 4.4 years. The mean VAS score was 3.2 ± 2.7 for pain at rest and 6.1 ± 2.5 for pain on activity. The mean HHS was 47.4 ± 13.0. The mean CSI score was 19.5 ± 11.3. The classification of CS severity level according to the scoring in CSI Part A is shown in Table 2. Seventy-nine patients were classified as subclinical (score: 0–29), 16 were classified as mild (score: 30–39), 3 were classified as moderate (score: 40–49), 2 were classified as severe (score: 50–59), and none were classified as extreme (score: 60–100). Five percent of the patients had a score of 40 or more points. Prevalence rates of CSS diagnoses according to CSI Part B are shown in Table 3.

**Table 1:** Patients’ demographics and clinical assessments (*N* = 100)

	Mean ± SD or *N* = %
Sex, N	Male:15, Female:85
Age (years)	63.9 ± 11.6
Tönnis grade, N	0:0 1:0 2:23 3:77
Duration of hip pain (years)	4.2 ± 4.4
VAS pain at rest	3.2 ± 2.7
VAS pain on activity	6.1 ± 2.5
HHS	47.4 ± 13.0
CSI score	19.5 ± 11.3

Note: All data are reported as mean ± standard deviation ratings, unless otherwise indicated

Abbreviations: *SD* standard deviation, *VAS* Visual Analogue Scale, *HHS* Harris Hip Score, *CSI* Central Sensitization Inventory

**Table 2:** The classification of CS severity level according to the CSI score

CSI score	*N* = %
Subclinical (0–29)	79
Mild (30–39)	16
Moderate (40–49)	3
Sever (50–59)	2
Extreme (60–100)	0
40 or above	5

Note: The percentage of patients by central sensitization severity levels and patients with a score of 40 points or higher in Central Sensitization Inventory Part A

Abbreviations: *CS* Central Sensitization, *CSI* Central Sensitization Inventory

**Table 3:** The prevalence rates of patients with a history of CSSs

CSS Diagnoses	*N* = %
Restless leg syndrome	0
Chronic fatigue syndrome	0
Fibromyalgia	0
Temporomandibular joint disorder	3
Migraine or tension headaches	3
Irritable bowel syndrome	3
Multiple chemical sensitivities	1
Neck injury including whiplash	4
Anxiety or panic attacks	1
Depression	5
Number of patients with at least one CSS	15

Note: The prevalence of patients with a history of each central sensitization syndrome (CSS) and at least one CSS in Central Sensitization Inventory Part B

Abbreviations: *CSSs* Central Sensitization Syndromes

### Relationship between radiographic hip OA severity and the CSI score

The CSI score did not differ significantly between patients with Tönnis grades 2 and 3 (*P* = 0.063) in Table 4. There were also no significant differences in other factors, including patient demographics and clinical scores.

**Table 4:** Comparison between hip OA severities and patients’ demographics and clinical assessments

	Tönnis grade 2 (*N* = 23)	Tönnis grade 3 (*N* = 77)	*P*-value
Male/Female, N	6/17	9/69	0.090
Age (years)	63.7 ± 12.4	64.0 ± 11.4	0.446
Duration of hip pain (years)	3.0 ± 2.5	4.9 ± 5.7	0.310
VAS pain at rest	2.9 ± 2.7	3.2 ± 2.8	0.717
VAS pain on activity	6.0 ± 2.8	6.1 ± 2.4	1.000
HHS	48.7 ± 14.1	47.1 ± 12.7	0.306
CSI score	15.3 ± 8.2	20.8 ± 11.8	0.063

Note: All data are reported as mean ± standard deviation, unless otherwise indicated. Continuous variables were calculated using the Mann-Whitney U test and categorical variables were calculated using the chi-square test. *P* values < 0.05 were considered to indicate statistical significance

Abbreviations: *VAS* visual analogue scale, *CSI* Central Sensitization Inventory, *HHS* Harris Hip Score

### Correlation between characteristics of hip OA patients and CSI score

**Fig. 2:**
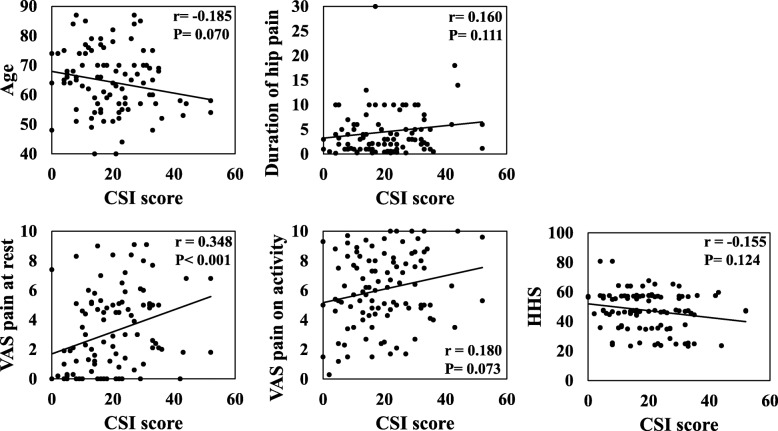
Correlation between the CSI score and characteristics of the hip OA patients. Correlations between the Central Sensitization Inventory score and age, duration of hip pain, visual analogue scale (VAS) pain at rest, VAS pain on activity, and Harris Hip Score in 100 patients with hip OA were evaluated using the Spearman’s correlation coefficient. Correlation coefficient is represented by r. *P* < 0.05 indicates statistical significance. Abbreviations: CSI: Central Sensitization Inventory; OA: Osteoarthritis; VAS: visual analogue scale; HHS: Harris Hip Score

The CSI score significantly correlated with VAS pain at rest (*r* = 0.348, *P* < 0.001) in all patients with hip OA (Fig. 2). Other factors, including age, duration of hip pain, VAS pain on activity, and HHS, were not significantly associated with the CSI score.

### The CSI score in hip OA patients with and without CSSs

Fifteen percent of the patients were diagnosed with one or more CSSs. The CSI score was significantly higher in patients with one or more CSSs (30.00 ± 12.50) than in patients without a CSS diagnosis (17.70 ± 10.00; *P* < 0.001) (Table 5). There were no significant differences in other factors, including patient demographics and clinical scores.

**Table 5:** Comparison between patients with and without CSSs in patients’ demographics and clinical assessments

	with CSSs (*N* = 15)	without CSSs (*N* = 85)	*P*-value
Male/Female, N	1/14	14/71	0.327
Age (years)	59.1 ± 13.1	64.8 ± 11.2	0.189
Duration of hip pain (years)	4.1 ± 3.0	4.5 ± 5.5	0.522
VAS pain at rest	4.2 ± 3.2	3.0 ± 2.6	0.120
VAS pain on activity	6.5 ± 2.8	6.0 ± 2.4	0.440
HHS	45.3 ± 10.1	47.8 ± 13.3	0.364
CSI score	30.0 ± 12.5	17.7 ± 10.0	**< .001**

Note: All data are reported as mean ± standard deviation, unless otherwise indicated. Continuous variables were calculated using the Mann-Whitney U test and categorical variables were calculated using the chi-square test. Statistically significant *P*-value (< 0.05) is in bold

Abbreviations: *CSSs* Central Sensitization Syndromes, *VAS* visual analogue scale, *CSI* Central Sensitization Inventory, *HHS* Harris Hip Score

## Discussion

A CSI score of 40 out of 100 was reported as the best distinguishing factor in patients with CSSs [13]. Kim et al. reported that 48% of knee OA patients who underwent total knee arthroplasty had a CSI score of 40 or higher and patients with high CSI scores (> 40) before knee arthroplasty exhibited more severe postsurgical pain intensity [16]. Patients with CSI scores of > 40 before vertebral fusion surgery exhibited higher (i.e. worse) patient-reported disability scores after the surgery [19]. In the present study, 5% of the patients with hip OA had CSI scores of at least 40 points and 15% of the patients had a history of at least one CSS. In addition, hip OA patients with CSSs had a higher CSI score compared to hip OA patients without CSSs. Although the percentage of patients with a score of 40 or more in this study was small compared to those of previous knee OA studies, it may be necessary to consider the presence of CS pathology according to different pain mechanisms in individuals undergoing pain management for hip OA, in particular, those with CSSs.

Previous studies have reported that a relationship exists between radiographic hip OA and pain severity, but have found no evidence of an association between preoperative pain severity and hip OA grade [20]. In our study, no relationship between hip OA severity and the CSI score was discovered; however, CSI scores were significantly correlated with pain at rest in hip OA patients. Pain on activity might be a common phenomenon, while pain at rest may sometimes occur with a variety of pain complaints (e.g., from a dull ache to a sharp, stabbing pain) [21]. Previous studies have suggested different associations of OA-related pain at rest and during activity in the knee and hip joints [22, 23]. Lundblad et al. reported an association between high preoperative VAS pain at rest and a lower pain threshold [23]. Taken together with previous studies’ results, and our results suggested that higher CSI score in hip OA patients was associated with the pathology of pain at rest.

Recently, some treatments of OA pain related to CS components have been reported [8–11]. Duloxetine is a potent selective serotonin norepinephrine reuptake inhibitor that potentiates the descending inhibitory pain pathways in the central nervous system [24]. The analgesic effect of duloxetine in patients with centrally mediated pain due to OA is well documented [10, 25]. From the results of this study, approaches to the pathology of CS may be important for hip OA patients with pain, particularly, pain at rest.

This study has a limitation to acknowledge. This study used a cross-sectional study design. The relationship between preoperative CS components and postoperative clinical results remains unclear in patients with hip OA. Therefore, a prospective longitudinal study will be needed to evaluate these relationships.

## Conclusions

In this study, 1 out of every 20 hip OA patients had CS components. CSI scores were significantly correlated with pain at rest in hip OA patients. Our results suggest that CS approach to hip OA may be one of the treatment options for pain at rest.

## Data Availability

The datasets supporting the conclusions of this article are included within the article. The raw data can be requested from the corresponding author.

## Abbreviations

OA: Osteoarthritis
CS: Central sensitization
VAS: Visual analogue scale
HHS: Harris Hip Score
CSI: Central Sensitization Inventory
CSS: Central sensitization syndrome
